# Gluten in Celiac Disease—More or Less?

**DOI:** 10.5041/RMMJ.10360

**Published:** 2019-01-28

**Authors:** Inna Spector Cohen, Andrew S. Day, Ron Shaoul

**Affiliations:** 1 Pediatric Gastroenterology & Nutrition Institute, Ruth Rappaport Children’s Hospital, Rambam Medical Center, Haifa, Israel; 2 Ruth & Bruch Rappaport Faculty of Medicine, Technion–Israel Institute of Technology, Haifa, Israel; 3 Department of Paediatrics, University of Otago, Christchurch, New Zealand

**Keywords:** Adherence, celiac, gluten, safety, small bowel

## Abstract

To date, the only known effective treatment for celiac disease is a strict gluten-free diet for life. We reviewed the literature to evaluate the upper limit for gluten content in food, which would be safe for patients with celiac disease. Patients with celiac disease should limit their daily gluten intake to no more than 10–50 mg. Most health authorities define gluten-free products as containing less than 20 parts per million gluten.

## INTRODUCTION

Celiac disease (CD) is an immune-mediated small intestinal enteropathy triggered by the ingestion of gluten in the genetically susceptible individuals, with prevalence of 1%–2% in North and South America, North Africa, Middle East, and India,^1^,^2^ although reports of 3% incidence in Denver^3^ and Sweden^4^ have emerged.

## WHAT IS GLUTEN?

Gluten is the name given to a group of wheat grain storage proteins found in wheat and related grains (rye and barley) abundantly consumed in Western diet, averaging 10–20 g per person/day. Gluten is composed of prolamins (gliadins in wheat, hordein in rye, and secalin in barley) and glutelins (glutenins in wheat).^5^ The prolamins are a complex group of alcohol-soluble proteins and constitute the major seed proteins in cereals and about 50% of the proteins in mature cereal grain. Other gluten proteins showing analogous immunogenic properties are present also in barley (hordeins), rye (secalins), oats (avenins), and other-closely related grains. These proteins are rich in proline and glutamine residues, making them resistant to gastrointestinal digestion and encouraging the deamination by tissue transglutaminase (tTG).^6^ Four fractions of gliadin have been described: α, β, γ, and ω subunits; the α-gliadin subunit has the most intense deleterious effects, while β, γ, and ω exert milder toxicity.^7^,^8^

The deamination of gliadin by tTG2 results in a complex with high affinity for the human leukocyte antigen (HLA) DQ2 or DQ8 pockets present in antigen-presenting cells. The release of pro-inflammatory cytokines such as interferon-γ and others maintain the pro-inflammatory events that result in functional deterioration of the mucosa (including intestinal permeability). During these processes, activation and release of metalloproteinases are responsible for the typical architectural changes, with flattening of villi, increased intraepithelial lymphocytes, hypertrophy of crypts, and increased cellularity (mainly lymphocytes and plasmocytes) in the lamina propria.^9^

To date, the only known effective treatment for celiac disease (CD) is a strict gluten-free diet (GFD) for life, excluding dietary wheat, rye, barley, and hybrids like kamut and triticale.^9^ In the majority of patients, lifelong strict GFD improves histological lesions, blood biochemistry, clinical manifestations (such as osteoporosis, anemia, depression, and infertility, and growth failure and delayed puberty in children^5^,^10^), mortality,^5^ and the risk of CD-related complications (malignancy, intestinal lymphoma).^5^,^11^ Untreated patients have 2- to 4-fold increased risk of non-Hodgkin’s lymphoma, more than 30-fold increased risk of small intestinal adenocarcinoma, and 1.4-fold increased risk of death.^12^ Furthermore, there are reports which suggest that the prevalence of autoimmune diseases among patients with CD is proportional to the duration of exposure to gluten.^13^

## GLUTEN-FREE DIET

Typically, GFD is based on a combination of foods naturally lacking gluten that are unlikely to be contaminated and special products formulated with gluten-free grains, usually labeled as “gluten-free.”^9^ With all its advantages, a GFD is difficult to adhere to since gluten is hidden in many industrially processed foods, including “gluten-free” products that may be potentially contaminated. Nevertheless, gluten-free products can be cross-contaminated.^14^ Gluten can also be found in pharmaceutical products^15^ and religious ceremony foods.^16^ Strict GFD restricts the patient’s social and school/working activities, inducing significant effects on the patient’s quality of life.^17^ The most common cause of non-response is failure to adhere to the prescribed GFD, either voluntarily or unintentionally.^18^ Many non-compliant patients are asymptomatic and have normal hematologic and biochemical lab results, despite the presence of notable mucosal villous atrophy and inflammation, which further limits compliance.^19^ There is a long ongoing debate regarding the safe amount of daily gluten consumption to avoid clinical and histological damage in celiac patients. Quite a few studies have focused on this topic.

Ciacci and her colleagues^11^ followed 390 celiac adults for at least 2 years during treatment of celiac disease with a gluten-free diet. The amount of ingested gluten was not mentioned. They noted that intestinal damage at follow-up was present in only 56.4% of patients and anti-endomysial antibodies (EMA) were present in the serum of 24.9% of patients. After a statistical analysis they showed that laboratory and clinical information has a high positive predictive value for the identification of patients with intestinal damage, and a low negative predictive value for the absence of intestinal damage even when the damage is severe. Jansson et al.^20^ performed gluten challenge over a 12-month period in 54 children with CD who had been on a gluten-free diet for at least 12 months. The patients were allotted to receive either 0.2 or 0.5 g/kg/d of gluten. Fifty-one patients (94%) relapsed within the first 4 weeks and 100% after 8 weeks of gluten challenge. In this study there was a gluten dose-dependency for severity of the enteropathy induced during 4 weeks of gluten challenge.

Catassi et al.^21^ showed that the effects of chronic ingestion of a small amount of gliadin in children with CD were dose-dependent. They administered 100 or 500 mg gliadin/day (200 mg or 1 g gluten/ day, respectively) to celiac children during 4 weeks and found that those receiving 100 mg reported no clinical symptoms and had minimal intestinal mucosal changes, while those who received 500 mg had pronounced mucosal damage and three out of ten patients reported anorexia and pale stools. In a later study, Catassi with other colleagues performed a prospective, randomized, double-blind, placebo-controlled study^22^ in which 39 CD patients were divided into three intervention groups receiving 10 mg or 50 mg of gluten or placebo. Results showed that 50 mg gluten/d, if introduced for 3 months, was sufficient to cause a significant worsening of the small intestine villous height/crypt depth (VH/CrD) ratio in treated CD patients. Interestingly, seven of thirteen patients who consumed 10 mg of gluten per day also had worsening of their VH/CrD. Allowing for the morphological changes, there was no significant change in IgA anti-tissue transglutaminase antibodies, and even a significant decrease in anti-gliadin (AGA) IgG antibodies was noted. These findings were contrary to a study which found no relationship between the presence or absence of villous atrophy and ingestion of a gluten-free diet.^23^ This study compared patients with CD on a Codex-GFD (a diet based on the Codex Alimentarius Commission of the World Health Organization that allows up to 0.03% protein derived from gluten-containing grains to be included in so-called gluten-free foods, principally in the form of wheat starch and malt) to CD patients that were on a non-gluten detectable GFD. The proportions of patients with a normal biopsy specimen or villous atrophy did not differ between groups consuming either a non-gluten detectable GFD or a Codex-GFD. Moreover, intraepithelial lymphocyte (IEL) counts in those with a normal specimen were also similar in each dietary group.

In another study, Troncone et al.^24^ showed significant changes in small-bowel mucosa in four out of six adolescents with CD that were taking 0–500 mg gluten daily (higher number of crypt intraepithelial lymphocytes and increased crypt volumes, diminished volume of villous epithelium), with AGA IgA and EMA positive in only one of them.

Collin et al.^25^ estimated daily gluten intake from 4-day food records in 76 adults and 16 children with celiac disease adhering to a strict gluten-free diet for 1–10 years; 28 adults were taking naturally gluten-free, 48 adults and all children consumed wheat starch-based gluten-free products. They found no significant correlation between the use of flours and intestinal mucosal histology in adults. In children, the mean daily use of flours was 60 g (range: 20–140 g); they all had normal small-bowel morphology at follow-up.

Ciclitira et al.^26^ showed that 10 mg gliadin by intraduodenal infusion over 8 hours did not change the jejunal mucosa in one patient with celiac disease, while 100 mg gliadin produced minor changes. This group also showed that, after 1 week, seven adult celiac patients receiving between 1.2 and 2.4 mg gliadin from gluten-free bread in addition to GFD without any commercial gluten-free products exhibited a significant reduction in the mean VH/ CrD.^27^ Nevertheless, the same group of researchers later found no significant difference in jejunal morphometry in a 6-week period during which patients consumed between 1.2 mg and 2.4 mg of gluten from bread compared to another 6-week period where this product was not ingested.^28^

Kaukinen et al.^29^ showed that in 52 adults and children the mucosal integrity (VH/CrD and enterocyte heights) was unrelated to the daily gluten intake of between 5 and 150 mg gluten daily (mean 34 mg) for about 8 years. IgA-class (EMA) or antireticulin antibodies (ARA) were not present in any of these patients, and three had positive AGA titers despite normal small-bowel mucosal architecture.

Lohiniemi et al.^30^ also reported that adult celiac patients consuming an average of 36 mg of gluten per day (range 0–180 mg) did not develop clinical symptoms. In this study the villous architecture was normal in 21 of 23 small-bowel biopsies, while one had subtotal and another partial villous atrophy.

On the other hand, in a study conducted by Chartrand and her colleagues,^19^ a much smaller dose of gluten (1.5 mg daily) ingested for a 1-year period triggered persistent symptoms in 11 of 17 patients and intermittent symptoms in another five. Another interesting finding was that gluten consumption of this amount did not influence the titers of IgA or IgG AGA. Moreover, even in the group with long-term consumption of the same amount of gluten (average 6 years) there were low titers of AGA and negative EMA titers. Mucosal histology was not assessed in this study.

Laurin et al.^31^ performed a gluten challenge that lasted 5–51 weeks. Children with CD were instructed to ingest 10 g of gluten daily, but the actual intake, as was noted in their diary records, was 20–260 mg daily. They found no correlation between the gluten intake and the time course for clinical symptoms. Nevertheless, in 22 of 23 patients the IEL count increased after challenge, with positive correlation noted between gluten intake and IEL numbers in the post-challenge biopsies. In this study 90% of children developed specific antibodies after 2 months.

Mayer and his colleagues followed adolescents diagnosed with CD.^32^ The patients were classified to those on strict GFD, those on normal gluten-containing diet, and patients on gluten-free diet with occasional intake of small amounts of gluten ranging from 0.06 to 2 g/day (average 0.73 g/day). Nine of 14 patients ingested less than 1 g of gluten per day. On morphometric analysis small intestinal mucosa was normal in all biopsies of patients on a gluten-free diet, showed structural changes in 11 of 14 patients on a semi-strict gluten-free diet (from considerable villous shortening to flat mucosa), while all but one subjects on a gluten-containing diet had flat, or severely damaged, small intestinal mucosa. Another finding was that in the group on a semi-strict gluten-free diet neither symptoms nor antigliadin antibody concentrations were found to be reliable markers of gluten ingestion. Biagi et al. studied the long-term effect of small amounts of gluten consumption. They showed that the ingestion of 1 mg of gluten per day over a period of 2 years by a woman with CD (by intake of a small fragment of communion wafer) prevented histological recovery.^16^

Several review papers tried to determine the safe daily gluten consumption: Hischenhuber et al. in a review article suggested that allowed gluten intake in patients with CD should be between 10 and 100 mg/day.^33^

Akobeng et al. in their review article suggested that daily gluten intake of <10 mg is unlikely to cause significant histological abnormalities.^34^

A health hazard assessment for gluten exposure conducted by the US FDA in 2011 concluded that the tolerable daily intake level for gluten in individuals with CD for chronic exposure is 7.0 mg gluten/day for adverse morphological effects and 0.015 mg gluten/day for adverse clinical effects.^35^

## WHAT CAN BE LABELED AS A GLUTEN-FREE PRODUCT?

Gibert and her colleagues collected data on gluten-free products consumption by celiac patients in Mediterranean countries (Italy and Spain) and Northern countries (Norway and Germany).^36^ They suggested defining as gluten-free any product with a gluten content of less than 20 parts per million (ppm).

Dessi et al. in their review article concluded, based upon current data, that wheat starch-based food is safe, provided it contains <100 mg gluten/kg.^5^

Codex Alimentarius which represents a consensus of international standards for food safety, established in 2008 that the cutoff for “gluten-free” products was 20 ppm (milligrams of gluten per kilogram of product).^37^

Many countries have now set local cutoffs, ranging from 20 ppm in Spain, Italy, UK,^37^ Canada, and USA, to 10 ppm in Argentina and 3 ppm in Australia, New Zealand, and Chile.^9^

The European Union (EU) gluten-free legislation published in 2009 and regulated in 2012 specifies two subgroups: gluten-free (≤20 ppm/mg/kg) and low gluten (21–100 ppm/mg/kg).^38^

Since gluten-free products made with wheat starch do contain small amounts of immunodetectable gluten, the label gluten-free should be considered a misnomer.^19^

Although the CD antibody tests show a high accuracy for selecting patients needing a diagnostic biopsy, these tests do not seem to be reliable after diagnosis as the autoantibody titers do not correlate well with histological findings or symptoms in CD patients on a GFD.^19^,^22^,^23^,^29^,^32^,^39^–^47^

## CONCLUSION

There seems to be no clear consensus on the safe amount of daily gluten intake. This may be related to differences between individuals, method of gluten intake assessment, and differences in methodology between the different studies. Total daily gluten consumption that seems to be safe for most CD patients is <50 mg gluten; nevertheless, some CD patients need as little as 10 mg of daily gluten to promote development of intestinal mucosal abnormalities. It should be noted that in assessing intake with food diaries, there is always a risk of over- or underestimating the real intake. Until new data will be available the recommendation for children should be a gluten-free diet (less than 20 ppm).

## Abbreviations

CD: celiac disease
EMA: anti-endomysial antibodies
GFD: gluten-free diet
IEL: intraepithelial lymphocyte
ppm: parts per million
tTG: tissue transglutaminase
VH/CrD: villous height/crypt depth ratio.
